# Oral Rinse versus Facemask Capture for Nonsputum Diagnosis of Pulmonary Tuberculosis

**DOI:** 10.1164/rccm.202506-1477RL

**Published:** 2025-09-12

**Authors:** Simon C. Mendelsohn, Gabriella T. Jackson, Edmund Wessels, Elizabeth Beyers, Suzette Visagie, Marcia Steyn, Sudesh Sivarasu, Bavesh D. Kana, Thomas J. Scriba, Mark Hatherill, for the Facemask Study Team, Hadn Africa

**Affiliations:** ^1^ South African Tuberculosis Vaccine Initiative, Institute of Infectious Disease and Molecular Medicine and Division of Immunology, Department of Pathology, University of Cape Town, Cape Town, South Africa;; ^2^ Biomedical Engineering Research Centre, Department of Human Biology, University of Cape Town, Cape Town, South Africa; and; ^3^ School of Pathology, University of Witwatersrand, Johannesburg, South Africa

*To the Editor*:

Delays in pulmonary tuberculosis (TB) diagnosis contribute to continued *Mycobacterium tuberculosis* (Mtb) transmission (1). Earlier treatment may reduce TB morbidity, mortality, and socioeconomic burden. TB is primarily diagnosed through passive case finding, relying on symptomatic individuals seeking care, typically using sputum-based diagnostics at health facilities. This has limitations: High-quality sputum often depends on supervision by trained healthcare workers; and many individuals delay seeking care because of indirect costs such as travel, time off from work, and long clinic wait times (2). Simple, noninvasive collection methods are urgently needed for mass screening. During the coronavirus disease (COVID-19) pandemic, the feasibility and acceptance of self-testing and home-based sample collection were demonstrated with easily obtainable specimens, such as nasal or oral swabs (3). Likewise, non–sputum-based TB tests—particularly those able to detect Mtb in both symptomatic and asymptomatic individuals—could expand access to early diagnosis and treatment, reaching people whose cases were missed by sputum-based tools.

Two promising nonsputum Mtb sampling methods, oral rinse (4, 5) and facemask capture (6, 7), may enable self-collection at home or in community settings, supporting broader, earlier TB case detection (8). However, more evidence is needed to inform assay optimization and interpretation before these approaches might inform TB screening policy. To our knowledge, no previous study has directly compared them using the commercially available GeneXpert MTB/RIF Ultra (Ultra) platform (Cepheid). We conducted a head-to-head comparison of oral rinse and facemask capture for the diagnosis of pulmonary TB, evaluating performance by symptom status and sputum Mtb burden, to inform their potential roles in passive and active TB case finding.

Some of the results of these studies have been previously reported in the form of a preprint (VeriXiv, June 16, 2025; https://doi.org/10.12688/verixiv.1306.1).

In a nested case-control study in Worcester, South Africa, we recruited HIV-negative adults (⩾18 yr old) with newly diagnosed, bacteriologically confirmed pulmonary TB from clinics and community control participants without acute disease in the prior month. No participants had a TB diagnosis within the previous 2 years. TB cases were excluded if they were diagnosed by means of a trace-positive Ultra result only or had received more than a single dose of TB treatment. Ethics approval was granted by the University of Cape Town Human Research Ethics Committee (248/2022). All participants provided written, informed consent.

Participants wore a modified duckbill N95 facemask with two polyvinyl alcohol patches—three-dimensionally printed in-house, each measuring 30 × 30 × 0.2 mm—for 1 hour during normal tidal breathing. Patches were removed with sterile forceps. One patch was immediately dissolved in 2 ml molecular-grade water in a sterile homogenization bag by manual manipulation. Participants then rinsed their mouths for 1 minute with 5 ml sterile water, supervised by study staff. There was no specific preprocedure preparation, and participants were not fasted. Each sample type was mixed with Xpert SR buffer (Cepheid) in a ratio of 0.75:1.5 ml and tested on-site with Ultra. Thereafter, sputum samples were collected for Mycobacteria Growth Indicator Tube culture and Ultra. The microbiological reference standard (MRS) was defined as sputum Mtb positivity by both culture and Ultra. Individuals who tested negative according to both were classified as control participants. Those with a single discordant or missing culture or Ultra result were excluded from analysis. Symptomatic and asymptomatic TB were defined as microbiologically confirmed TB with or without any reported symptoms (cough, fever, weight loss, night sweats, pleuritic chest pain, fatigue, or hemoptysis) of any duration, respectively. We calculated sensitivity and specificity using standard methods. We calculated 95% confidence intervals (CIs) using the Wilson method. McNemar’s test was used for paired proportions.

**Figure 1.**
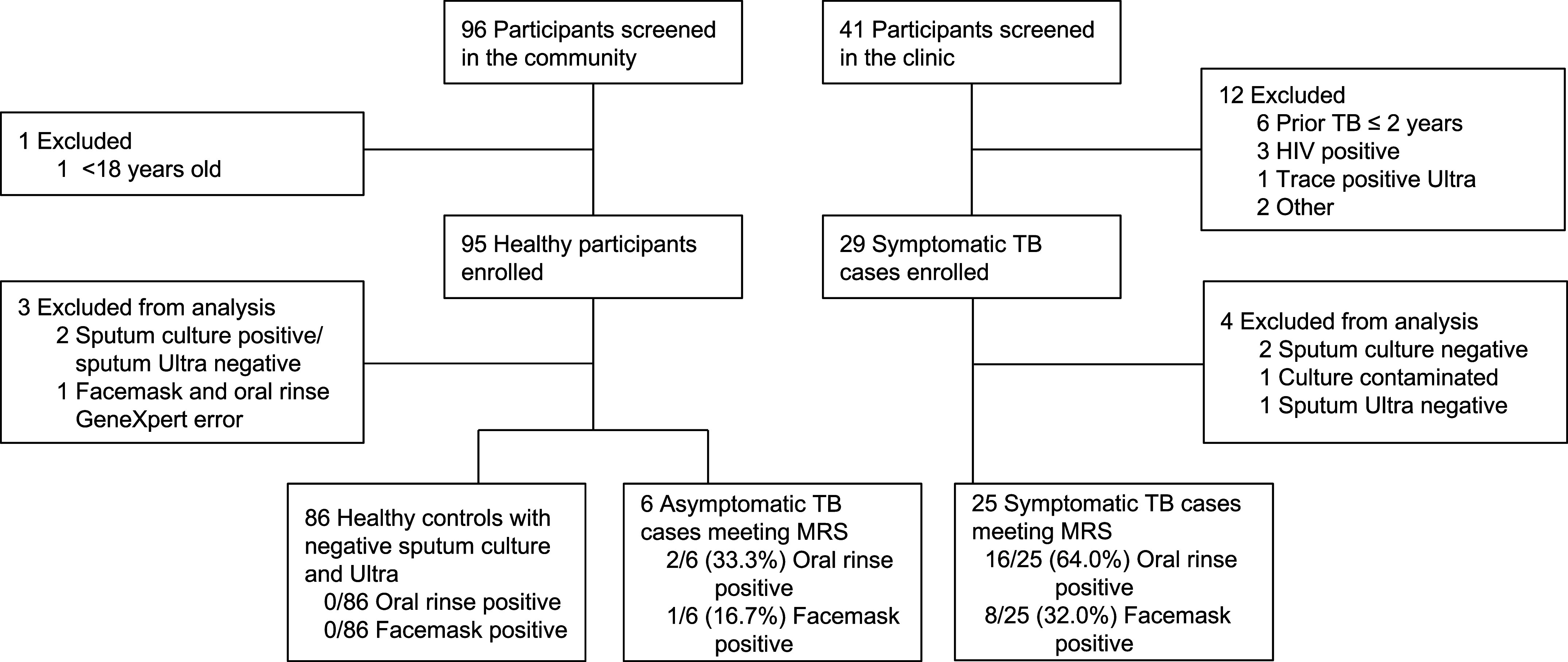
Participant flow. MRS = microbiological reference standard; TB = tuberculosis.

Between October 14, 2024, and May 5, 2025, 137 participants were screened and 124 were enrolled, including 29 participants with symptomatic TB from the clinic and 95 healthy participants from the community (Figure 1). Four individuals with TB and three healthy individuals were excluded from analyses for not meeting the MRS.

Among 25 symptomatic clinic attendees with TB, oral rinse sensitivity (*n* = 16; 64.0%; 95% CI = 44.5–80.0) was higher (absolute difference, 32%; 95% CI = 5.8–58.2; *P* = 0.0047) than facemask capture sensitivity (*n* = 8; 32.0%; 95% CI = 17.2–52.0). Specificity among 86 healthy community control participants without TB was 100% (*n* = 86; 95% CI = 95.7–100) for both methods. Among 92 community participants, 6 (6.5%) asymptomatic individuals were sputum positive for the MRS. Among the 6 individuals with asymptomatic TB who were identified through community screening, oral rinse sensitivity was 33.3% (*n* = 2; 95% CI = 9.7–70.0) versus 16.7% (*n* = 1; 95% CI = 3.0–56.0) for facemask capture.

**Figure 2.**
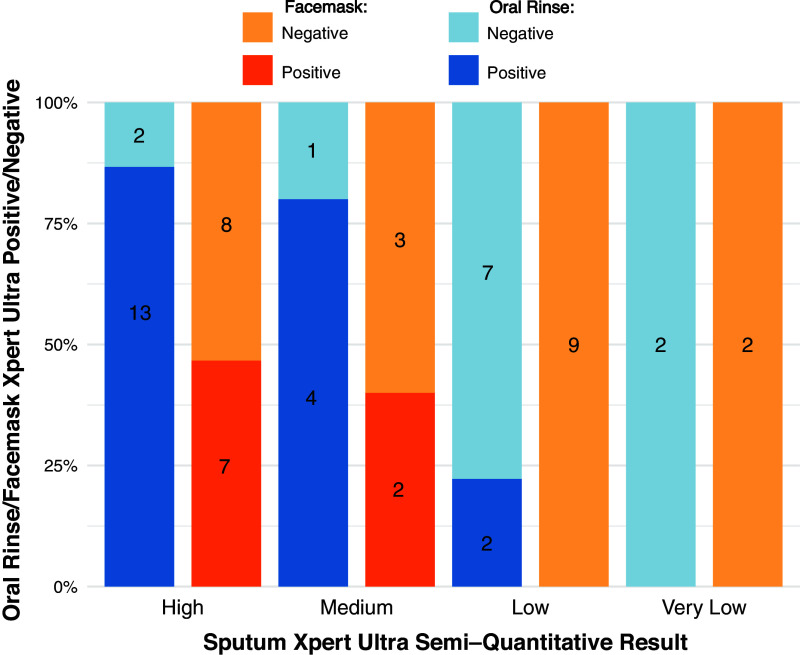
Oral rinse and facemask Xpert MTB/RIF Ultra positivity by sputum Xpert MTB/RIF Ultra semiquantitative grade among all tuberculosis cases.

Sensitivity of oral rinse and facemask capture among all TB cases with sputum semiquantitative Xpert Ultra graded medium–high (*n* = 20) was 85.0% (*n* = 17; 95% CI = 64.0–95.0) and 45.0% (*n* = 9; 95% CI = 25.8–66.0), respectively (Figure 2); for those graded very low–low (*n* = 11), sensitivity was 18.2% (*n* = 2; 95% CI = 5.1–48.0) and 0.0% (*n* = 0; 95% CI = 0.0–26.0), respectively.

Oral rinse sampling was twice as sensitive as facemask capture for pulmonary TB diagnosis by Xpert Ultra. Similarly, oral rinse sensitivity for symptomatic TB was double that for asymptomatic TB; and among all TB cases, sensitivity for those with moderate-to-high sputum Mtb bacillary burden was double that for cases with low bacillary burden. According to the latest World Health Organization Target Product Profiles for TB screening tests, highly specific (⩾98%) nonsputum tests with lower sensitivity (⩾60%) can still have programmatic utility with high population coverage (9, 10). A rapid, noninvasive sampling methodology is likely to pose less aerosolization risk than sputum expectoration and may, therefore, reduce infection control risk to healthcare workers and other patients. Home- or community-based collection and repeated sampling might reduce overall diagnostic loss, despite potential reductions in sensitivity (9, 10). This tradeoff may be particularly acceptable in settings where barriers to clinic access and sputum production limit the effectiveness of traditional, passive TB case finding. However, our findings suggest that oral rinse may have limited diagnostic performance for detecting asymptomatic or sputum-paucibacillary TB, which could constrain its role in active case-finding settings. We hypothesize that better sensitivity in high sputum bacillary burden cases may be due to increased bacterial shedding into the oral cavity because of greater Mtb load in the respiratory tract.

This study has certain limitations. First, the sample size is small; however, it is suitable for a hypothesis-generating pilot study of a novel approach. Second, findings may not be generalizable to populations such as people living with HIV, children, or individuals with recent prior TB, all of whom were excluded from this study. Third, the use of a healthy community control group may overestimate specificity; future studies should prospectively enroll individuals with presumptive TB. Fourth, no sample processing optimization was undertaken, and specimens were tested directly in the Xpert Ultra cartridge. Methodological refinements to oral rinse sampling could improve diagnostic sensitivity. Our findings support the continued optimization and evaluation of oral rinse as a simple, rapid, screening tool to expand TB screening beyond the constraints of facility-based sputum collection.
